# TRAIL (CD253) Sensitizes Human Airway Epithelial Cells to Toxin-Induced Cell Death

**DOI:** 10.1128/mSphere.00399-18

**Published:** 2018-09-26

**Authors:** Yinghui Rong, Jennifer Westfall, Dylan Ehrbar, Timothy LaRocca, Nicholas J. Mantis

**Affiliations:** a Division of Infectious Disease, Wadsworth Center, New York State Department of Health, Albany, New York, USA; b Department of Basic and Clinical Sciences, Albany College of Pharmacy and Health Sciences, Albany, New York, USA; University of Kentucky

**Keywords:** bioterrorism, cytokines, epithelial cells, inflammation, lung defense, toxins

## Abstract

Inhalation of ricin toxin is associated with the onset of acute respiratory distress syndrome (ARDS), characterized by hemorrhage, inflammatory exudates, and tissue edema, as well as the nearly complete destruction of the lung epithelium. Here we report that the Calu-3 human airway epithelial cell line is relatively impervious to the effects of ricin, with little evidence of cell death even upon exposure to microgram amounts of toxin. However, the addition of exogenous soluble tumor necrosis factor (TNF)-related apoptosis-inducing ligand (TRAIL; CD253) dramatically sensitized Calu-3 cells to ricin-induced apoptosis. Calu-3 cell killing in response to ricin and TRAIL exposure was partially inhibited by caspase-8 and caspase-3/7 inhibitors, consistent with involvement of extrinsic apoptotic pathways in cell death. We employed nCounter Technology to define the transcriptional response of Calu-3 cells to ricin, TRAIL, and the combination of ricin plus TRAIL. An array of genes associated with inflammation and cell death were significantly upregulated upon treatment with ricin toxin and were further amplified upon addition of TRAIL. Of particular note was interleukin-6 (IL-6), whose expression in Calu-3 cells increased 300-fold upon ricin treatment and more than 750-fold upon ricin and TRAIL treatment. IL-6 secretion by Calu-3 cells was confirmed by cytometric bead array analysis. On the basis of these finding, we speculate that the severe airway epithelial cell damage observed in animal models following ricin exposure is a result of a positive-feedback loop driven by proinflammatory cytokines such as TRAIL and IL-6.

**IMPORTANCE** Ricin toxin is a biothreat agent that is particularly damaging to lung tissue following inhalation. A hallmark of ricin exposure is widespread inflammation and concomitant destruction of the airway epithelium. In this study, we investigated the possible interaction between ricin and known proinflammatory cytokines associated with lung tissue. Using an established human airway epithelial cell line, we demonstrate that epithelial cell killing by ricin is significantly enhanced in the presence of the proinflammatory cytokine known as TRAIL (CD253). Moreover, epithelial cells that are simultaneously exposed to ricin and TRAIL produced large amounts of secondary proinflammatory signals, including IL-6, which in the context of the lung would be expected to exacerbate toxin-induced tissue damage. Our results suggest that therapies designed to neutralize proinflammatory cytokines such as TRAIL and IL-6 may limit the bystander damage associated with ricin exposure.

## Abstract (precis)

Ricin toxin is a biothreat agent that is particularly damaging to lung tissue following inhalation. A hallmark of ricin exposure is widespread inflammation and concomitant destruction of the airway epithelium. In this study, we investigated the possible interaction between ricin and known proinflammatory cytokines associated with lung tissue. Using an established human airway epithelial cell line, we demonstrate that epithelial cell killing by ricin is significantly enhanced in the presence of the proinflammatory cytokine known as TRAIL (CD253). Moreover, epithelial cells that are simultaneously exposed to ricin and TRAIL produced large amounts of secondary proinflammatory signals, including IL-6, which in the context of the lung would be expected to exacerbate toxin-induced tissue damage. Our results suggest that therapies designed to neutralize proinflammatory cytokines such as TRAIL and IL-6 may limit the bystander damage associated with ricin exposure.

## INTRODUCTION

NATO’s Biomedical Advisory Council recently concluded that ricin ranks at the top of the list of potential biothreat agents, due in large part to the toxin’s extreme potency against numerous different cell types as well as to its capacity to be disseminated via aerosol (1). In rodents, swine, and nonhuman primates (NHPs), inhalational ricin exposure evokes what is clinically equivalent to acute respiratory distress syndrome (ARDS) (2–4). In rodents and NHPs, the 50% lethal dose (LD_50_) of ricin by aerosol is ∼4 μg/kg of body weight (5–7). The hallmarks of ricin-induced lung damage include early (h 6 to h 12) onset of alveolar macrophage apoptosis followed hours later by intra-alveolar edema, accumulation of inflammatory cytokines in bronchoalveolar lavage (BAL) fluid, neutrophilic infiltration, and fibrinous exudate (5, 7–10). Airway epithelial cells are also a primary target of ricin intoxication and may play a role in amplifying toxin-induced pathology through secretion of proinflammatory cytokines and chemokines (5, 7, 11, 12).

Ricin itself is a potent inducer of apoptosis (13). The toxin is derived from castor beans (Ricinus communis), where it accumulates in storage vesicles as a mature, 65-kDa glycosylated protein (14–16). Ricin’s two subunits, RTA and RTB, are joined by a single disulfide bond. RTB is a galactose- and N-acetylgalactosamine (Gal/GalNAc)-specific lectin that promotes ricin attachment to cell surface glycoproteins and glycolipids and facilitates ricin’s retrograde transport to the endoplasmic reticulum (ER). RTA is an RNA N-glycosidase (EC 3.2.2.22) that catalyzes the hydrolysis of a conserved adenine residue within the sarcin/ricin loop (SRL) of 28S rRNA (13, 17, 18). In the ER, RTA is liberated from RTB and is retrotranslocated via the Sec61 complex into the cytoplasm, where it inactivates ribosomes with great efficiency (17–19). Programmed cell death in alveolar macrophages and primary human bronchial epithelial cells occurs via caspase-3-dependent mechanisms, although the exact upstream signals (e.g., ribosome inactivation, ribotoxic stress, and mitogen-activated protein kinase [MAPK] and NFκb signaling) are yet to be elucidated.

The goal of the current study was to better define the response of airway epithelial cells to the effects of ricin, especially in light of recent quantitative analyses of ribosomal depurination status that indicated that the pulmonary epithelial cells are disproportionately affected by ricin following intranasal challenge (11). Specifically, we reasoned that cells already compromised by ricin would be hypersensitive to secondary insults such as those represented by proinflammatory cytokines which are known to accumulate in the bronchoalveolar lavage (BAL) fluid of animals following a ricin inhalation, especially the early response cytokines interleukin-1 (IL-1) and tumor necrosis alpha (TNF-α) (2, 8, 12, 20–24). TNF-α and related cytokines such as TRAIL (tumor necrosis factor-related apoptosis-inducing ligand; CD253) are suspected to have a role in driving lung epithelial cell death considering their established capacities to trigger extrinsic apoptotic cell death in different cell types experiencing intracellular stress from another insult (25).

## RESULTS

### TRAIL sensitizes Calu-3 cells to ricin toxin-induced death.

Ricin is a promiscuous toxin capable of killing virtually all mammalian cell types. In most cases, >50% cell death occurs within 12 to 48 h of exposure of cells to nanogram amounts of ricin (26). We chose Calu-3 cells as a model to understand the response of human airway epithelial cells to ricin toxin. Calu-3 cells are a lung adenocarcinoma line widely accepted as a model to study drug and nanomaterial interactions with pulmonary epithelium (27–31). Calu-3 cells have also been used as a model to assess transcytosis of botulinum toxin (32) and mucosal IgA (33), as well as infection by respiratory pathogens (34).

**FIG 1:**
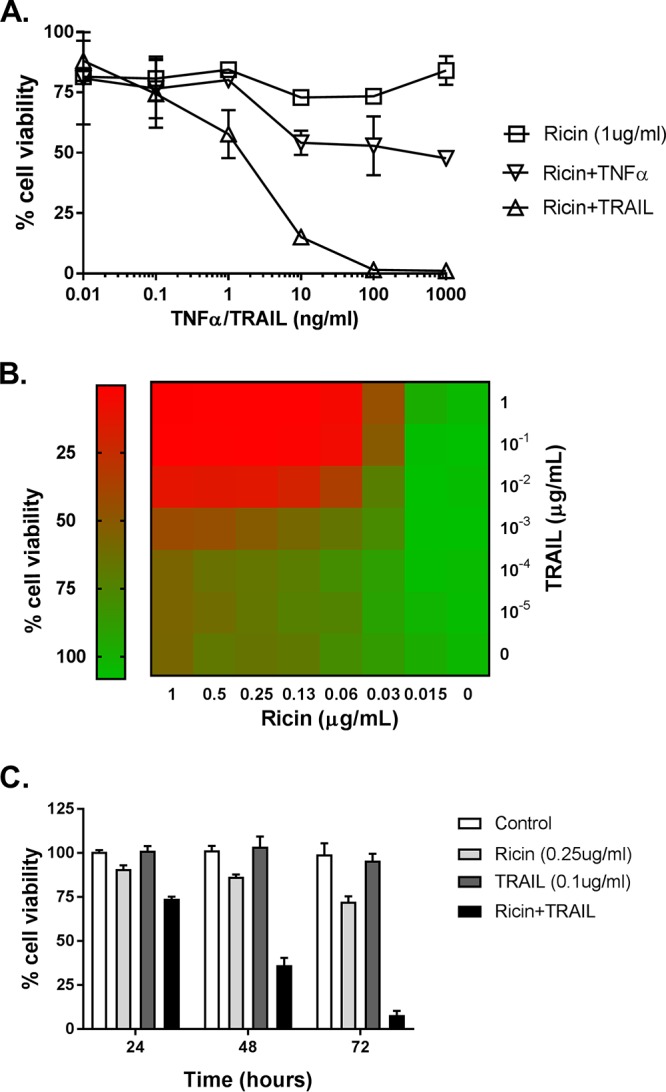
The sensitizing effect of TRAIL on ricin-induced cell death in Calu-3 cells. (A) TNF-ɑ or TRAIL (starting at 1 µg/ml) in a 10-fold serial dilution was mixed with ricin (1 µg/ml) and then administered to the cells seeded in 96-well plates for 24 h. The cells were then washed, and cell viability was measured 72 h later, as described in Materials and Methods. (B) In the dose experiment, cell viability was assessed at 72 h after exposure of the cells to the indicated concentrations of ricin and TRAIL. (C) In the time course experiments, cell viability was assessed 24 h, 48 h, or 72 h after the cells were exposed to ricin (0.25 µg/ml) and TRAIL (0.1 µg/ml). All treatments were performed in triplicate and repeated 3 times. Viability of 100% was defined as the average value obtained from wells in which cells had been treated with medium only.

**FIG 2:**
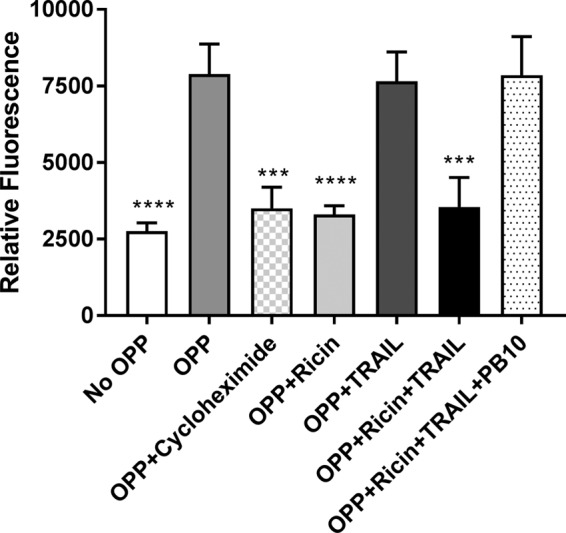
Protein synthesis in Calu-3 cells following treatments with ricin and/or TRAIL. Calu-3 cells were plated at a density of 5 × 10^4^ cells/well in a 96-well clear-bottom black plate. After confluence was achieved, cells were treated with ricin (0.25 µg/ml) or TRAIL (0.1 μg/ml) or a mixture of ricin and TRAIL with or without anti-ricin MAb PB10 (15 µg/ml) for 24 h. As a control, cells were treated with cycloheximide (250 µg/ml). Cells were then incubated for 2 h in culture medium alone, culture medium containing OPP, or culture media containing OPP and the treatments as described above. The cells were then processed for detection of protein synthesis, as described in Materials and Methods. The results (means ± standard deviations [SD]) represent a single experiment done in triplicate and repeated twice. ***, *P*  < 0.001; ****, *P*  < 0.0001 (versus only OPP-treated cells).

To assess the sensitivity of Calu-3 cells to ricin toxin, nearly confluent Calu-3 cells grown in microtiter plates were treated with a range of ricin toxin doses (0.08 to 10 μg/ml) and then assessed for viability 24 to 72 h later. We found that Calu-3 cell viability was largely unaffected by ricin, as determined using an ATP-based cell viability assay (Fig. 1A; see also Fig. S1 in the supplemental material). However, under these conditions, protein synthesis was inhibited to a level comparable to that achieved with cycloheximide treatment, indicating that the cells were indeed affected by exposure to ricin but that cell viability remained high (Fig. 2). Calu-3 cell monolayers grown on Transwell filters were similarly insensitive to killing by ricin, although transepithelial resistance (TER) declined significantly following ricin treatment, indicating that the toxin impacted epithelial integrity (Fig. S2). To determine whether lung epithelial cell lines are generally more resistant to ricin-induced cell death than other cell types, we performed parallel studies with A549 cells, another established human lung epithelial cell line. The viability profile of A549 cells exposed to ricin resembled that of Calu-3 cells (Fig. S3) (35). The issue was not related to the potency of ricin, as exactly the same lot of toxin was used in numerous Vero cell studies and mouse lethal challenge studies, as reported in separate manuscripts (36, 37). Thus, we conclude that two well-established human lung epithelial cell lines are hyposensitive to ricin-induced cell death.

10.1128/mSphere.00399-18.1 FIG S1 The effect of ricin on cell viability in Calu-3 cells. Cells seeded in 96-well plates were treated with ricin with the indicated concentrations (measured in micrograms per milliliter) for 24 h. The cells were then washed, and cell viability was measured 72 h later, as described in Materials and Methods. All treatments were performed in triplicate, and 100% viability was defined as the average value obtained from wells in which cells were treated with medium only. Download FIG S1, JPG file, 0.0 MB. Copyright © 2018 Rong et al. 2018 Rong et al. This content is distributed under the terms of the Creative Commons Attribution 4.0 International license.

10.1128/mSphere.00399-18.2 FIG S2 Effect of TRAIL on ricin-induced cell death in polarized Calu-3 cells grown on Transwell filters. Calu-3 cells were seeded at a density of 5 × 10^5^cells/ml into the upper chamber (0.1 ml) of the Transwell cell culture supports. Medium (0.5 ml) was added to the lower chamber. The medium was replaced on both sides every 2 days. Formation of contiguous cell monolayers was evaluated by microscopic examination following medium replacement. On day 12, polarized cells were treated with ricin (1 µg/ml) or TRAIL (1 µg/ml) or a ricin and TRAIL mixture or medium alone in the apical compartment for 24 h. (A) Development of tight junctions was monitored by measuring the transepithelial electrical resistance (TEER) every 1 to 2 days. (B) Three days after ricin treatment (on day 15), cell viability was measured. All treatments were performed in triplicate, and 100% viability was defined as the average value obtained from wells in which the cells had been treated with medium only. Download FIG S2, JPG file, 0.0 MB. Copyright © 2018 Rong et al. 2018 Rong et al. This content is distributed under the terms of the Creative Commons Attribution 4.0 International license.

10.1128/mSphere.00399-18.3 FIG S3 Effect of TRAIL on ricin-induced cell death of A549 cells. A549 cells were seeded at 1.2 × 10^4^/well into 96-well plates. After 24 h, A549 cells were treated with ricin (0.01 µg/ml) or TRAIL (0.1 µg/ml) or a mixture of ricin and TRAIL or medium only (negative control) for 24 h. Cell viability was assessed using CellTiter-GLO reagent. All treatments were performed in triplicate and repeated 3 times. Viability of 100% was defined as the average value obtained from wells in which cells were treated with medium only. Download FIG S3, JPG file, 0.0 MB. Copyright © 2018 Rong et al. 2018 Rong et al. This content is distributed under the terms of the Creative Commons Attribution 4.0 International license.

Lung epithelial cell death *in vivo* in response to infectious agents such as influenza virus is typically driven by local inflammatory responses. Therefore, we reasoned that proinflammatory cytokines such as TNF-α, which is known to be released by alveolar macrophages in response to ricin, might sensitize Calu-3 cells to toxin-induced cell death (8). TRAIL (Apo2L; CD253) is another proinflammatory cytokine of interest, considering its role in accelerating lung epithelial cell death under conditions of ARDS (38, 39). We therefore examined the viability of Calu-3 cells following treatment with a fixed amount of ricin (1 μg/ml) plus recombinant TNF-α or TRAIL at doses ranging from 0.01 ng/ml to 1,000 ng/ml (Fig. 1A). Calu-3 cells treated with 1 μg/ml of ricin alone displayed ∼20% cell death at the 72-h time point. The addition of 10 ng/ml of TNF-α resulted in slight increase in cell killing (50% to 60% cell death), although further increasing the amount of the TNF-α did not further exacerbate ricin’s cytotoxic activity. TRAIL was significantly more potent than TNF-α in promoting the enhancement of ricin-induced Calu-3 cell death. We observed virtually 100% cell death when cells were treated with ricin and ≥100 ng/ml TRAIL. Neither TNF-α (data not shown) nor TRAIL (Fig. 2) by itself had an effect on protein synthesis in Calu-3 cells. To see if TRAIL had a similar effect on other cell epithelial airway cells, we treated A549 cells with ricin or TRAIL or the combination of ricin and TRAIL (Fig. S3). Cell viability was greatly reduced when the cells were treated with the combination of ricin and TRAIL (∼90% cell death), compared to control (untreated) cells. TRAIL alone did not impact cell viability, whereas ricin (10 ng/ml) induced ∼25% cell death. Thus, TRAIL sensitizes lung epithelial cells to the effects of ricin.

**FIG 3:**
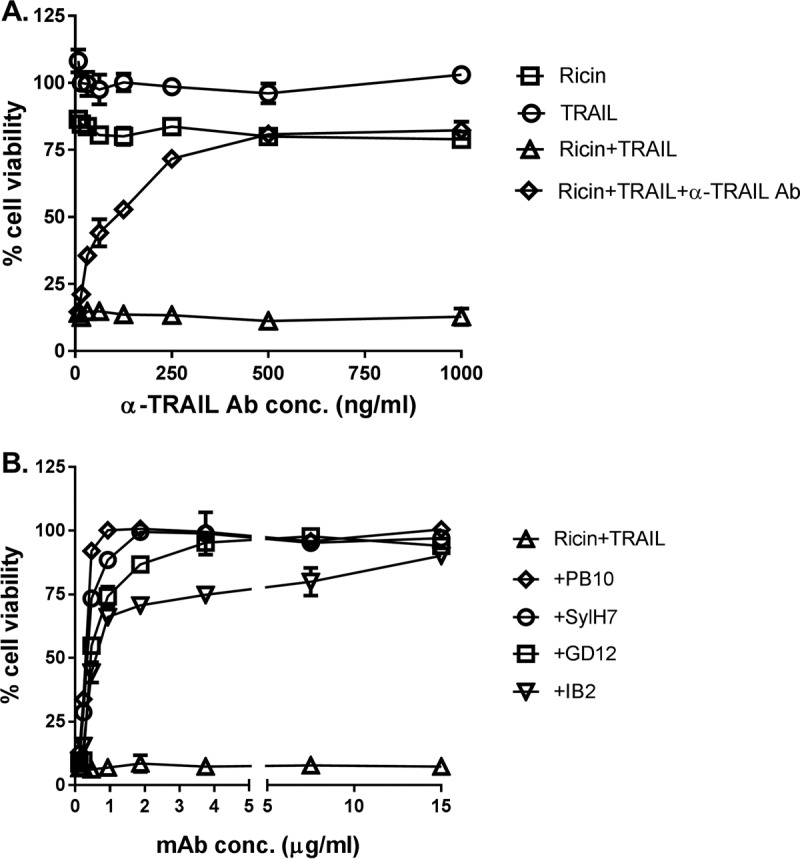
Specificity of ricin and TRAIL in inducing Calu-3 cell death. (A) Anti-TRAIL Abs (starting at 1 µg/ml) or (B) anti-ricin MAbs (starting at 15 µg/ml) in a 2-fold serial dilution were mixed with ricin (0.25 µg/ml) and TRAIL (0.1 µg/ml) and then administered to the cells seeded in 96-well plates for 24 h. The cells were then washed and cell viability was measured 72 h later, as described in Materials and Methods. The results (means ± SD) represent a single experiment done in triplicate and repeated at least three times.

To better define the observed synergy between ricin and TRAIL, we performed checkerboard analysis across a range of concentrations of ricin (0 to 1 µg/ml) and TRAIL (0 to 1 µg/ml) (Fig. 1B). This analysis identified the minimal doses of ricin (250 ng/ml) and TRAIL (100 ng/ml) required to achieve ∼100% cell death within a 72-h period. A time course of Calu-3 cell viability in response to ricin (250 ng/ml) or TRAIL (100 ng/ml) or the combination of ricin and TRAIL is shown in Fig. 1C. The viability of ricin-treated Calu-3 cells declined only marginally within a 72-h period, while the viability of the TRAIL-treated cells was largely unchanged in that same time frame. In contrast, the cell viability of Calu-3 cells treated with the combination of ricin and TRAIL declined in a stepwise manner at 24 h, 48 h, and 72 h to <25%. The observed effects of ricin and TRAIL on Calu-3 cell death and protein synthesis arrest were annulled by anti-TRAIL antibodies (Abs) (Fig. 3A) or neutralizing monoclonal antibodies (MAbs) against ricin’s A subunit (Fig. 3B) or B subunit (Fig. S4). The 50% inhibitory concentrations (IC_50_s) for each of the RTA and RTB neutralizing MAbs in conjunction with the Calu-3 cells were similar to what has been reported for other cell types such as Vero cells and THP-1 cells (36, 40). For example, MH3’s IC_50_ for Calu-3 cells was ∼ 1 μg/ml, which is similar to what we reported for MH3 on Vero cells (1.6 μg/ml) (36).

10.1128/mSphere.00399-18.4 FIG S4 Toxin-neutralizing activity of anti-RTB MAbs. The MAbs (starting at 30 µg/ml) at a 2-fold serial dilution were mixed with ricin (0.25 µg/ml) and TRAIL (0.1 µg/ml) and then administrated to the cells seeded in 96-well plates for 24 h. The cells were then washed, and cell viability was measured 72 h later. The results (means ± SD) represent a single experiment done in triplicate and repeated at least three times. Download FIG S4, JPG file, 0.0 MB. Copyright © 2018 Rong et al. 2018 Rong et al. This content is distributed under the terms of the Creative Commons Attribution 4.0 International license.

**FIG 4:**
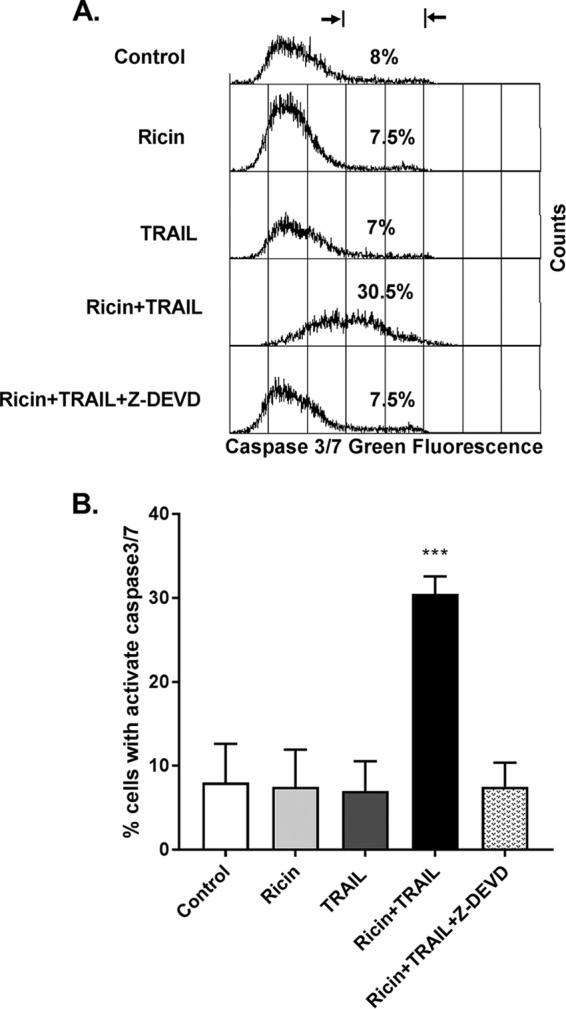
Increased caspase-3/7 activity in ricin- and TRAIL-treated Calu-3 cells. For the quantification of caspase-3/7 activity, Calu-3 cells were treated with ricin (0.25 µg/ml) or TRAIL (0.1 µg/ml) or ricin and TRAIL with or without caspase 3 inhibitor Z-DEVD-FMK(62.5 nM) for 24 h or with medium only (negative control). The caspase-3/7 activity levels were determined by flow cytometry as described in Materials and Methods. Caspase-3/7 activity was expressed as a percentage of total cells. The results are presented as means ± SD of three independent experiments. ***, *P*  < 0.001 (versus control cells).

**FIG 5:**
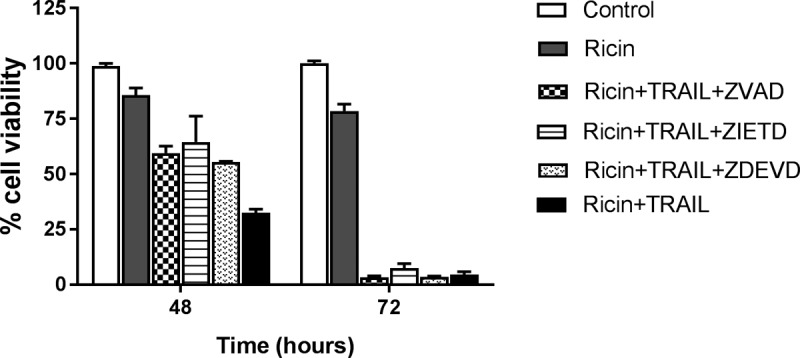
Protective effect of caspase inhibitors on cell viability in ricin- and TRAIL-treated Calu-3 cells. Pan-caspase inhibitor Z-VAD-FMK, caspase-3 inhibitor Z-DEVD-FMK, or caspase-8 inhibitor Z-IETD-FMK at 62.5 nM was mixed with ricin (0.25 µg/ml) and TRAIL (0.1 µg/ml) and administered to the cells for 24 h. The cells were then washed, and cell viability was measured 48 h or 72 h later. The results (means ± SD) represent a single experiment done in triplicate and repeated at least three times.

In macrophage and epithelial cells, ricin triggers the intrinsic apoptotic pathway through a process dependent on caspase-3/7 activation (13). We therefore examined caspase-3/7 activity in Calu-3 cells following treatment with ricin (250 ng/ml) or TRAIL (100 ng/ml) or the combination of ricin and TRAIL. At the concentrations employed, neither ricin nor TRAIL alone was sufficient to induce caspase-3/7 activity in Calu-3 cells (Fig. 4A). However, the combination of ricin and TRAIL resulted in a significant (∼4-fold) increase in caspase-3/7 activity, which was inhibited by Z-DVEVD (Fig. 4). In a Calu-3 cell viability assay, ZVAD (pan-caspase inhibitor), ZIETD (caspase-8 inhibitor), and ZDEVD (caspase-3/7 inhibitor) were each able to partially suppress the cytotoxic effects of ricin and TRAIL, but only at early time points (Fig. 5). Blocking the initiator caspase-9 with the inhibitor LEHD had no effect on Calu-3 cell viability following ricin and TRAIL treatment (Fig. S5), nor did treatment with the necrosis inhibitors necrosulfonamide (NSA), glycogen synthase kinase (GSK), and Nec-1 (Fig. S5). Collectively, these results are consistent with ricin and TRAIL treatment activating apoptosis through multiple caspase-8- and caspase-3/7-dependent pathways.

10.1128/mSphere.00399-18.5 FIG S5 Effects of caspase-9 inhibitor and necrosis inhibitors on cell viability in ricin- and TRAIL-treated Calu-3 cells. Cells were treated with ricin (0.25 µg/ml) and TRAIL (0.1 µg/ml) with or without caspase-9 inhibitors Z-LEHD-FMK (62.5 nM) (A) or necrosis inhibitors (NSA, GSK, or Nec-1; 6.25 µM) (B). After 24 h of incubation, cells were washed and cell viability was measured 24 h later. The results (means ± SD) represent a single experiment done in triplicate and repeated at least three times. Download FIG S5, JPG file, 0.0 MB. Copyright © 2018 Rong et al. 2018 Rong et al. This content is distributed under the terms of the Creative Commons Attribution 4.0 International license.

### Transcriptional profiling of Calu-3 cells following ricin and TRAIL treatment.

**FIG 6:**
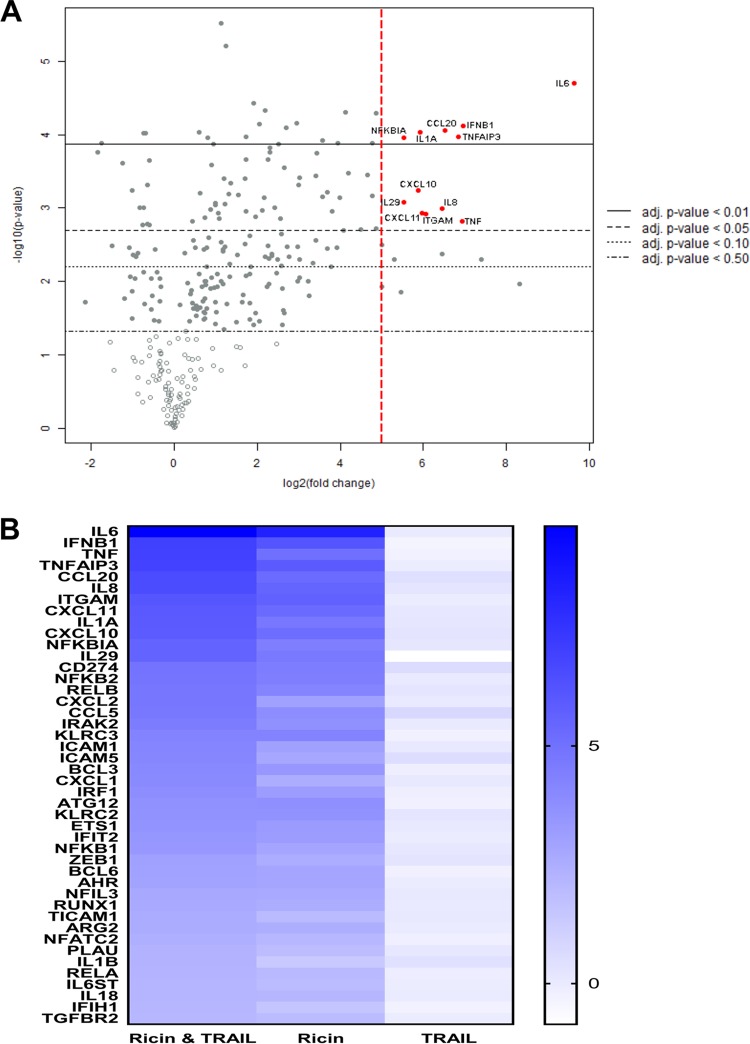
NanoString analysis of genes differentially expressed in ricin- and TRAIL-treated Calu-3 cells. Calu-3 cells were treated with ricin (0.25 µg/ml) or TRAIL (0.1 µg/ml) or a mixture of ricin and TRAIL or medium only (negative control) for 18 h. RNA was extracted and subjected to nCounter analysis using a human immunology array panel. (A) Volcano plot representation of gene expression changes in ricin-plus-TRAIL-treated cells, compared with control cells. Red circles represent transcripts upregulated >32-fold (5 log^2^-fold). The vertical dashed red line marks the 5 log^2^-fold change threshold. (B) Heat map showing the relative fold changes in expression of selected genes in each treatment group compared to the control group. Genes were selected based on a minimum of 4-fold (2 log^2^-fold)-higher expression in comparisons between control and ricin-plus-TRAIL-treated cells. The color scale bar denotes maximum counts in blue and minimal counts in white. The measured fold change values (relative to control cells) are listed in Table S1.

To better understand the interaction between ricin and TRAIL, we subjected Calu-3 cells to transcriptional profiling using a human immunology nCounter array encompassing ∼600 target genes. RNA was isolated from Calu-3 cells treated with ricin (250 ng/ml) or TRAIL (100 ng/ml) or the combination of ricin and TRAIL for 3 h, 6 h and 18 h. At the 3-h and 6-h time points, there were no significant changes in RNA levels seen among the target genes represented on the human immunology array in comparisons of samples with ricin or TRAIL or ricin-plus-TRAIL treatments to medium control samples (data not shown). By 18 h, the picture was markedly different. Analysis of the RNA from Calu-3 cells treated with the combination of ricin and TRAIL indicated that there were ∼80 genes whose expression was elevated >2-fold over the levels seen with the untreated controls, which corresponds to roughly 12% of all the genes on the human immunology nCounter array (Fig. 6; see also Table S1 and Fig. S6 in the supplemental material). Most notable was an increase in the level of IL-6 (∼750-fold), followed by other proinflammatory cytokines such as beta interferon (IFN-β) (∼120-fold), TNF-α (∼120-fold), IL-8 (88-fold), IL-1α (60-fold), and CCL20 (90-fold). Virtually the same transcriptional profile was observed when Calu-3 cells were treated with just ricin, although the magnitude of the response was dampened compared to that seen with cells treated with ricin plus TRAIL (Fig. 6; see also Table S1 and Fig. S6). TRAIL treatment alone did not significantly alter Calu-3 gene expression. These results are consistent with TRAIL enhancing the proinflammatory and apoptotic responses of Calu-3 to ricin rather than inducing parallel or convergent proinflammatory and apoptotic pathways.

10.1128/mSphere.00399-18.6 FIG S6 Transcriptional profiles of Calu-3 cells treated with ricin and TRAIL. Scatter plots show pair-wise comparisons of normalized log2-transformed gene expression values in Calu-3 cells. Each point represents the expression of a single gene for both groups shown on the graph. Genes with significantly different levels of expression between groups are indicated in colors; upregulated genes are colored in red and downregulated genes in blue. Linear regression analysis of the gene expression values between the groups was performed. Regression lines and Pearson correlation coefficients (*r*) are shown with associated *P* values. The 95% confidence interval is indicated with light gray shading. (A to C) Positive correlations between the control group and (A) ricin (*r* = 0.91, *P* = <2.2e−16), (B) ricin plus TRAIL (*r* = 0.89, *P* = <2.2e−16), and (C) TRAIL (*r* = 0.99, *P* = <2.2e−16) groups were observed. (D) A positive correlation between ricin and ricin plus TRAIL was also observed (*r* = 0.99, *P* = <2.2e−16). Low-count genes were included in this analysis as noted in the “Statistical analyses” section. Download FIG S6, JPG file, 0.1 MB. Copyright © 2018 Rong et al. 2018 Rong et al. This content is distributed under the terms of the Creative Commons Attribution 4.0 International license.

10.1128/mSphere.00399-18.7 TABLE S1 Transcriptional profiling of Calu-3 cells upon treatment with ricin plus TRAIL (18 h). Download Table S1, DOCX file, 0.0 MB. Copyright © 2018 Rong et al. 2018 Rong et al. This content is distributed under the terms of the Creative Commons Attribution 4.0 International license.

**FIG 7:**
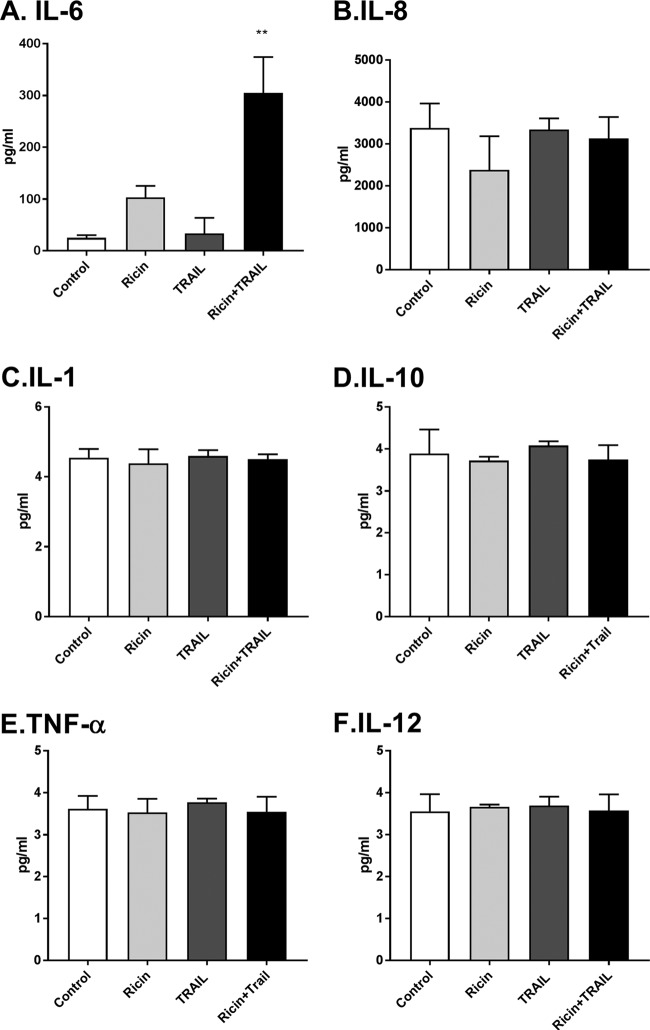
Cytokine secretion by Calu-3 cells following ricin and TRAIL treatment. Calu-3 cells were treated with ricin (0.25 µg/ml) or TRAIL (0.1 µg/ml) or a mixture of ricin and TRAIL or medium only (negative control) for 24 h. Cell supernatants were collected from treated cells. The levels of cytokines IL-6, IL-8, IL-1β, IL-10, TNF-ɑ, and IL-12p70 (panels A to F, respectively) were measured by CBA. The results are presented as the means ± SD of three independent experiments. **, *P*  < 0.01 (versus untreated cells [negative control]).

To validate the transcriptional profiling studies, Calu-3 cells were treated for 24 h with ricin or TRAIL or the ricin-plus-TRAIL combination, after which culture supernatants were assayed for levels of proinflammatory cytokines IL-6, IL-8, IL-1, IL-10, TNF-α, and IL-12 by cytometric bead array (CBA). We found that the IL-8, IL-1, IL-10, TNF-α, and IL-12 levels were unchanged, irrespective of whether Calu-3 cells were treated with ricin or TRAIL or the ricin-plus-TRAIL combination (Fig. 7). IL-6 levels, in contrast, were elevated >10-fold in supernatants from Calu-3 cells treated with ricin plus TRAIL, compared to controls (Fig. 7). Treatment of cells with ricin alone enhanced IL-6 levels, although not to a statistically significant degree. Thus, treatment of Calu-3 cells with the combination of ricin plus TRAIL results in the preferential secretion of IL-6.

**FIG 8:**
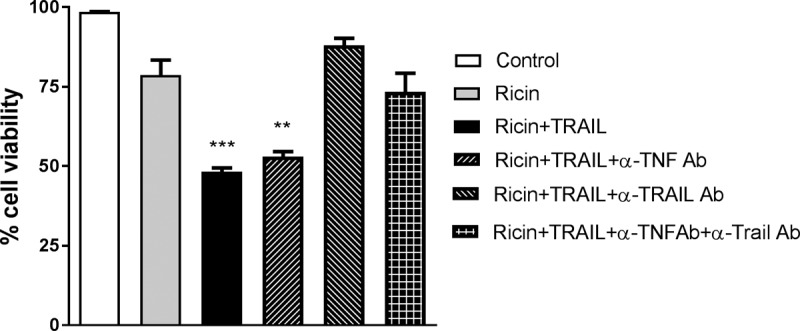
Effects of TNF-α- and TRAIL-neutralizing Abs on the viability of Calu-3 cells following ricin and TRAIL treatment. Calu-3 cells were treated with ricin or ricin plus TRAIL in the presence of neutralizing anti-TNF-α Ab or anti-TRAIL Ab or the combination of the two Abs. Cell viability was measured 72 h later. The results (means ± SD) represent a single experiment done in triplicate and repeated at least three times. **, *P*  < 0.01; *** *P*  < 0.001 (versus untreated cells [negative control]).

We postulated that the absence of TNF-α in the Calu-3 cell supernatants following ricin and TRAIL treatments might have been due to autocrine signaling such that soluble cytokine was rapidly captured by the TNF-α receptor, which in turn might have contributed to ricin-induced cell death. To examine this possibility, Calu-3 cells were treated with ricin plus TRAIL in the presence of neutralizing anti-TNF-α antibody. We found that anti-TNF-α antibody treatment did not prevent or even reduce Calu-3 cell killing in response to ricin plus TRAIL, whereas treatment with an anti-TRAIL neutralizing antibody did rescue the cells (Fig. 8).

## DISCUSSION

Widespread damage to the airway epithelium is a hallmark of inhalational ricin exposure, although the exact molecular events that culminate in epithelial cell destruction have not been fully elucidated (2, 3, 5, 7, 24, 41). For this study, we utilized the well-characterized Calu-3 cell line as a prototype to better define the response of human airway epithelial cells to ricin (27–31). We found that Calu-3 cells, when grown to confluence on solid or permeable substrates, were largely impervious to the effects of ricin-induced cell death (as determined using an ATP-based cell viability assay), even though protein synthesis was effectively shut down (as determined using an O-propargyl-puromycin [OPP]-based assay) and epithelial integrity compromised (as manifested by a reduction in TER). Coadministration of soluble TRAIL (and, to a lesser degree, TNF-α) rendered Calu-3 cells >1,000-fold more sensitive to toxin-induced apoptosis. Soluble TRAIL also magnified the proinflammatory transcriptional response of Calu-3 cells to ricin and the preferential secretion of IL-6. While the current investigation was limited to *in vitro* studies, the results are consistent with a model in which proinflammatory cytokines such as TRAIL amplify epithelial stress-induced signal transduction that promotes the recruitment of polymorphonuclear leukocytes (PMNs) to the lung and simultaneously lowers the threshold level of ricin required to induce epithelial apoptosis. We also present evidence to support the notion that ricin alters bronchial epithelial integrity and barrier function, thereby contributing to enhanced transudation into the alveolar space (42).

TRAIL has previously been implicated in driving respiratory pathology and airway epithelial cell death in mice and humans in response to pathogenic agents, notably, influenza virus, respiratory syncytial virus (RSV), and chlamydia (38, 39, 43–45). In the case of influenza virus infection, alveolar macrophages are the primary source of TRAIL (38, 39). Bronchial epithelial cells express a TRAIL receptor(s), which ultimately modulates TRAIL-dependent apoptosis (46). In rodents and nonhuman primates, alveolar macrophages are a primary target of ricin following intranasal and inhalational challenge, so it is plausible that these cells also serve as a source of TRAIL following toxin exposure (3, 5, 9, 47). It remains unclear whether this scenario applies to ricin. In preliminary studies, we have not yet observed a measurable uptick in TRAIL levels in the BAL fluids from mice or nonhuman primates that had been challenged with ricin (Y. Rong and N. Mantis, unpublished results). However, we have observed that supernatants from toxin-treated monocyte/macrophage cell lines do sensitize human lung epithelial cells to the effects of ricin, and we are actively examining whether this factor is TRAIL or a member of the TNF-α family of cytokines (C. Kempen, Y. Rong, N. Mantis, and T. LaRocca, unpublished results).

The issue of how TRAIL sensitizes Calu-3 cells to ricin-induced cell death is of particular interest, considering that TRAIL activates cell death through an extrinsic apoptotic pathway, whereas ricin triggers intrinsic pathways induced as a result of the ribotoxic stress response (RSR), unfolded protein response (UPR), and/or increased levels of intracellular calcium (13, 25). We postulate that caspase-3 serves as the central node through which ricin and TRAIL intersect. In human cells, activation of TRAIL receptor 1 (TRAIL-R1) and/or TRAIL-R2 stimulates caspase-8 activation, which in turn triggers caspase-3 activation (46). Ricin-induced programmed cell death is also dependent on caspase-3 activation, although which specific upstream signaling pathway(s) (e.g., RSR, UPR, ER stress) is most relevant in airway epithelial cell killing has not been completely elucidated (2, 12). As demonstrated in this study, Calu-3 cell death following with ricin and TRAIL treatment coincided with an increase in caspase-3/7 activity and was partially inhibited by the addition of Z-DEVD-fmk but was not impacted by LEHD, an inhibitor of caspase-9. Calu-3 cell death may also be exacerbated by a concomitant decline in the levels of endogenous inhibitors of apoptosis such as cFLIP, due to ricin’s capacity to arrest protein synthesis, as reported previously in a study of cells treated with Shiga toxin (48). In fact, the protein synthesis inhibitor cycloheximide is commonly used as a tool to sensitize cells to TRAIL-induced apoptosis (25, 49). While the issue is somewhat a question of semantics, we would argue that TRAIL sensitizes Calu-3 cells to ricin-induced apoptosis rather than sensitizing ricin-sensitizing Calu-3 cells to TRAIL-induced cell death. This claim is best supported by the results of the transcriptional profiling that we performed, which demonstrated that TRAIL amplifies the effects of ricin on the treatment of Calu-3 cells across the board. Treatment with TRAIL alone had no effect on Calu-3 gene expression, nor did TRAIL (by itself) negatively influence Calu-3 cell viability.

The relationship between protein synthesis arrest and apoptosis in response to ricin toxin is another interesting aspect of this study. Using the puromycin analogue OPP coupled with click chemistry to measure levels of protein synthesis (50), we found that ricin treatment induced protein synthesis arrest (i.e., a measurable decrease in ATP levels) in Calu-3 cells without triggering cell death. Only upon the addition of TRAIL did the cells undergo apoptosis. Thus, ricin-treated Calu-3 cells are primed to undergo programmed cell death upon exposure to a secondary insult, whether that insult is represented by TRAIL or possibly by other signals *in vivo* (25). As shown in Fig. 2 and 3 (see also Fig. S4 in the supplemental material), neutralizing antibodies against ricin or TRAIL protected Calu-3 cells from simultaneous ricin and TRAIL challenge. The relative kinetics of neutralizing activity were not investigated here. In other words, might anti-TRAIL antibodies have activity when administered after treatment of Calu-3 cells with ricin? Might the Calu-3 cell response to TRAIL be blunted with TRAIL receptor (e.g., DR2) antibodies? These are pertinent questions that are currently being addressed in a mouse model, even though the mouse differs considerably from humans with respect to TRAIL receptors (46).

We identified IL-6 as being markedly upregulated in Calu-3 cells at the transcriptional and protein levels following ricin and ricin-plus-TRAIL treatments. This finding may have important implications for understanding the pathology associated with pulmonary ricin exposure, especially in nonhuman primates, where elevated levels of IL-6 in bronchoalveolar lavage (BAL) fluids are associated with negative outcomes following toxin exposure (C. Roy, Y. Rong, D. Ehrbar, and N. Mantis, unpublished results). IL-6 also accumulates in BAL fluids and serum of mice following intranasal ricin challenge (Rong and Mantis, unpublished) (9, 22, 47, 51). DaSilva and colleagues also noted a significant increase (albeit modest) in IL-6 mRNA levels when total lung tissues from ricin-challenged mice were analyzed by DNA microarray (20). Whether IL-6 is more than just a biomarker of ricin intoxication remains to be determined, but there is considerable evidence to implicate this cytokine in driving local and systemic pathologies (52, 53). Surprisingly, Calu-3 cells did not secrete detectable levels of other “initiator” cytokines such as TNF-α and IL-1 or the chemokine IL-8, even though each of their respective mRNA transcripts was significantly unregulated by ricin or ricin plus TRAIL. Wong et al. noted that TNF-α secretion actually declined when primary human bronchial epithelial cells were exposed to ricin, presumably due to a global arrest in protein synthesis (12). The continued (and possibly preferential) synthesis of IL-6 in ricin-intoxicated cells may have to do with differential rates of mRNA stability, especially when mitogen-activated protein kinase (MAPK) signaling pathways are activated (54). It is worth noting that Wong and colleagues reported that TNF-α and IL-1β expression in primary human bronchial epithelial cells in response to ricin is dependent on NFκB, while IL-6 expression is not (12). In summary, our report advances our understanding of the factors that may drive the severe pathology associated with aerosolized ricin exposure and offers support for therapeutic approaches aimed at neutralizing ricin plus immunomodulatory factors such as TRAIL (2, 24).

## MATERIALS AND METHODS

### Chemicals and biological reagents.

Ricin toxin (Ricinus communis agglutinin II) was purchased from Vector Laboratories (Burlingame, CA). Ricin was dialyzed against phosphate-buffered saline (PBS) at 4°C in 10,000-molecular-weight (MW)-cutoff Slide-A-Lyzer dialysis cassettes (Pierce, Rockford, IL), prior to use in cytotoxicity studies. Fetal calf serum was purchased from Gibco-Invitrogen (Carlsbad, CA). Cells were maintained in a humidified incubator at 37°C with 5% CO_2_. Recombinant human tumor necrosis factor alpha (TNF-α), recombinant human soluble TRAIL (sTRAIL)/Apo2L/CD253, anti-human sTRAIL-(s)-Apo2L, and rabbit polyclonal anti-TRAIL antibody were purchased from Peprotech (Rocky Hill, NJ). Human TNF-α neutralizing rabbit Ab was purchased from Cell Signaling Technology (Danvers, MA). Z-LEHD-FMK, Z-VAD-FMK, Z-DEVD-FMK, and Z-IETD-FMK were purchased from ApexbBio (Taiwan). Necrostatin-1 (Nec-1), GSK′872, and necrosulfonamide (NSA) were purchased from EMD Millipore (Burlington, MA). Unless noted otherwise, all other chemicals were obtained from MilliporeSigma (St. Louis, MO). Murine MAbs against ricin toxin’s A subunit (PB10, SyH7, GD12, and IB2) and B subunit (24B11, SylH3, MH3, 8A1, 8B3, LF1, and LC5) were purified by protein A chromatography at the Dana Farber Cancer Institute (DFCI) Monoclonal Antibody Core facility (Boston, MA), as described previously (36, 55).

### Cell culture.

The Calu-3 lung adenocarcinoma cell line was obtained from the American Type Culture Collection (ATCC; Manassas, VA) and cultured in Eagle’s minimum essential medium (EMEM) supplemented with 10% fetal bovine serum, provided by the Wadsworth Center Media Services facility. Cells were grown in a humidified incubator containing 5% CO_2_ and 95% air at 37°C. The cells were plated in 75-cm^2^ cell culture flasks and subcultured at 70% to 90% confluence using a 0.25% trypsin–EDTA solution **(**Corning Life Sciences, Corning, NY). The culture medium was changed every 3 days. The cells were split 1:5 during each passage. The numbers of passages used for the following experiments were under 10. The A459 human lung epithelial cell line (ATCC CCL-185) was obtained from ATCC and maintained as recommended by ATCC.

### Ricin cytotoxicity assay.

Calu-3 and A549 cells were trypsinized, adjusted to 5 × 10^5^ cells per ml, seeded (100 μl/well) into 96-well plates (Corning Life Sciences, Corning, NY), and incubated for 3 to 4 days until confluence. Calu-3 cells were then treated with ricin, TRAIL, a mixture of ricin and TRAIL, or medium alone (negative control) for 24 h. The cells were washed to remove noninternalized toxin or TRAIL and were then incubated for 24 to 72 h. Cell viability was assessed using CellTiter-GLO reagent (Promega, Madison, WI) and a SpectraMax L microplate reader (Molecular Devices, Sunnyvale, CA). All treatments were performed in triplicate and repeated at least 3 times. Viability of 100% was defined as the average value obtained from wells in which cells were treated with medium only.

On the basis of the cytotoxicity results from the ricin and TRAIL treatments described above, ricin (0.25 µg/ml) and TRAIL (0.1 µg/ml) were used in all the subsequent experiments. To measure the neutralizing activity of ricin-specific MAbs and neutralizing anti-TRAIL Ab, the anti-RTA MAbs (starting at 15 µg/ml), anti-RTB MAbs (starting at 30 µg/ml), or anti-TRAIL Abs (starting at 1 µg/ml) in a 2-fold serial dilution were mixed with ricin and TRAIL and then administered to the cells seeded in 96-well plates for 24 h. After washing and then incubating for 3 days, cell viability was assessed using CellTiter-GLO reagent. Cell viability was normalized to cells treated with medium only. The protective effect of caspase inhibitors (Z-VAD-FMK, Z-LEHD-FMK, Z-DEVD-FMK, and Z-IETD-FMK), RIPK1 inhibitor (Nec-1), RIPK3 inhibitor (GSK′872), and MLKL inhibitor (NSA) was also evaluated using combinations with ricin and TRAIL and the concentrations and incubation times indicated for each experiment. Dimethyl sulfoxide (DMSO) was used as a control vehicle for all experiments.

### Protein synthesis assay.

Calu-3 cells were plated at a density of 5 × 10^4^ cells/well in a 96-well clear-bottom black plate (Corning Life Science, Corning, NY) and incubated for 3 to 4 days until confluence. Cayman’s protein synthesis assay kit (Cayman Chemical, Ann Arbor, MI) was used to measure the protein synthesis. Cells were treated with ricin (0.25 µg/ml), TRAIL (0.1 μg/ml), the mixture of ricin and TRAIL combined with PB10 (15 µg/ml), or cycloheximide (250 µg/ml; positive control) for 24 h. Cells were then incubated for 2 h in culture medium alone, culture medium containing O-propargyl-puromycin (OPP), or culture medium containing OPP plus the treatments described above. After fixing and washing, cells were incubated with 5 FAM-azide staining solution in the dark for 30 min for subsequent detection of OPP-labeled proteins. The cells were then examined immediately using a fluorescent plate reader (Becton, Dickinson, Franklin Lakes, NJ) set to excitation/emission at 485/535 nm.

### Caspase-3/7 activity assay.

For the quantification of caspase-3/7 activities after treatment, Calu-3 cells were labeled with 500 nM Cell Event caspase-3/7 green detection reagent (Invitrogen, Carlsbad, CA) for 30 min at 37°C in the dark. A total of 10,000 stained cells per sample were acquired and analyzed in a FACSCalibur flow cytometer by using CellQuest Pro software (Becton, Dickinson, Franklin Lakes, NJ). Data were expressed as a percentage of total cells.

### Multiplex gene expression analysis using NanoString.

Calu-3 cells were treated with ricin, TRAIL, the combination of ricin and TRAIL, or medium alone (negative control) for 24 h. RNA was extracted from treated or nontreated cells using an RNeasy Plus minikit with additional on-column DNase digestion with an RNase-Free DNase set (Qiagen Hilden, Germany). Protocols were followed according to the manufacturer's instructions. Extracted RNA samples were stored at −80°C until use. Upon assay, RNA integrity was verified by agarose gel electrophoresis. RNA quality and concentrations were measured using an Agilent 2100 Bioanalyzer (Life Technologies, Carlsbad, CA). RNA (100 ng) was hybridized with a predesigned nCounter human immunology panel that included 594 target genes and 15 internal reference genes. The geNorm algorithm was used to select the most stable of the reference genes (GAPDH [glyceraldehyde-3-phosphate dehydrogenase], PPIA, G6PD, EEF1G, GUSB, HPRT1, SDHA, and RPL19) used for normalization (56). The experimental procedures were carried out on a NanoString preparation station and digital analyzer according to the manufacturer's instructions. Two biological replicates were selected from each of the four groups for analysis.

### Cell supernatant cytokine quantification by cytometric bead array (CBA).

Cell supernatants were collected from treated Calu-3 cells. The BD CBA Human Inflammatory Cytokines kit (Becton, Dickinson, Franklin Lakes, NJ) was used to quantitatively measure levels of the following specific sets of cytokines: interleukin-8 (IL-8), interleukin-1β (IL-1β), interleukin-6 (IL-6), interleukin-10 (IL-10), TNF-α, and interleukin-12p70 (IL-12p70). Dilution series of human cytokine standards, included in the kit and prepared according to the manufacturer’s instructions, were employed in each assay run to enable quantification. Assays were performed according to the manufacturer’s instructions, and 50 μl of assay beads, 50 μl of the studied sample or standard, and 50 μl of phycoerythrin (PE)-labeled antibodies (Detection Reagent) were added consecutively to each sample tube and incubated at room temperature in the dark for 3 h. Next, the samples were washed and centrifuged, after which the pellet was resuspended in wash buffer and analyzed on the same day in a flow cytometer. Flow cytometry was performed using a four-laser BD FACSCalibur system utilizing BD CellQuest software for acquisition.

### Statistical analyses.

Statistical analyses were carried out using GraphPad Prism 7 (GraphPad Software, San Diego, CA), as well as the nCounter Advanced analysis module (v 1.1.4) of the nSolver Analysis software (v3). Differential expression of the genes examined was determined by multivariate linear regression, with group membership chosen as the predictor variable and binding density as a confounder. All *P* values derived from NanoString analysis were adjusted with Benjamini-Yekutieli correction for control of the false-discovery rate. Low-count genes were omitted using the default settings in the nCounter Advanced analysis software for all analyses except the linear regression of gene expression values.
