# Supplementary figures and images for: TRAIL (CD253) Sensitizes Human Airway Epithelial Cells to Toxin-Induced Cell Death
Source: mSphere. 2018 Sep 26;3(5):e00399-18. doi: 10.1128/mSphere.00399-18 (PMC6158510; doi:10.1128/mSphere.00399-18)

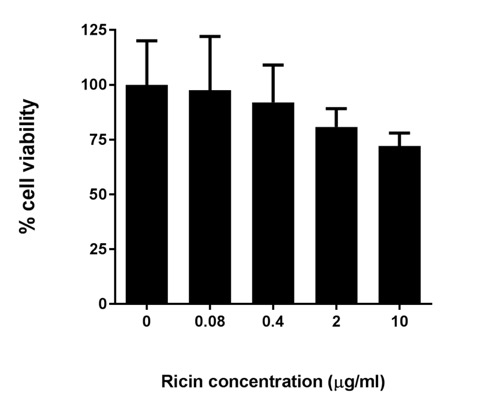

Supplement: FIG S1 [file sph005182652sf1.jpg]

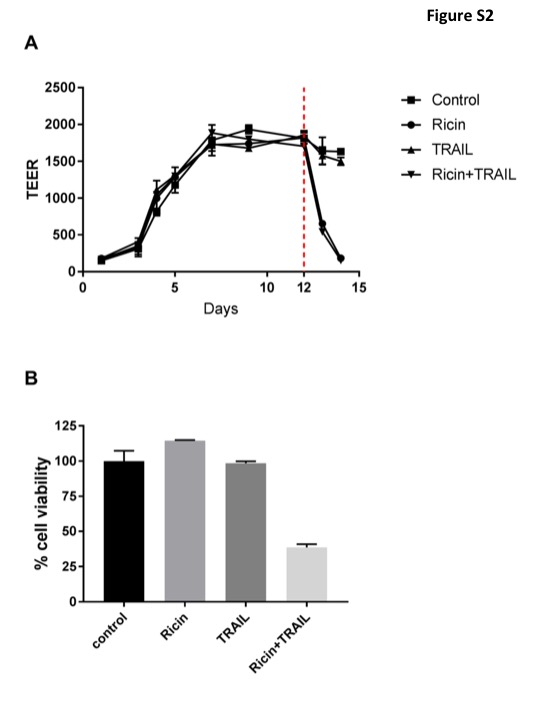

Supplement: FIG S2 [file sph005182652sf2.jpg]

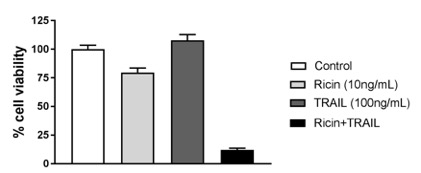

Supplement: FIG S3 [file sph005182652sf3.jpg]

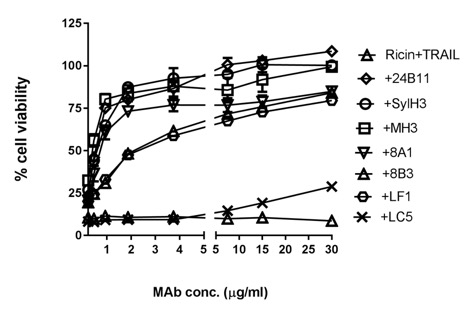

Supplement: FIG S4 [file sph005182652sf4.jpg]

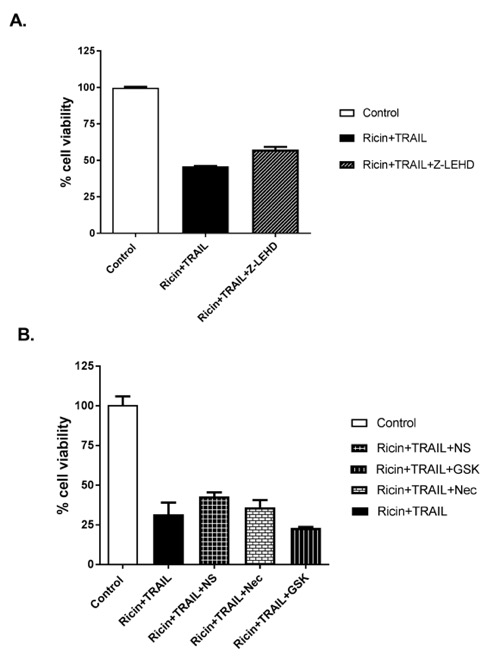

Supplement: FIG S5 [file sph005182652sf5.jpg]

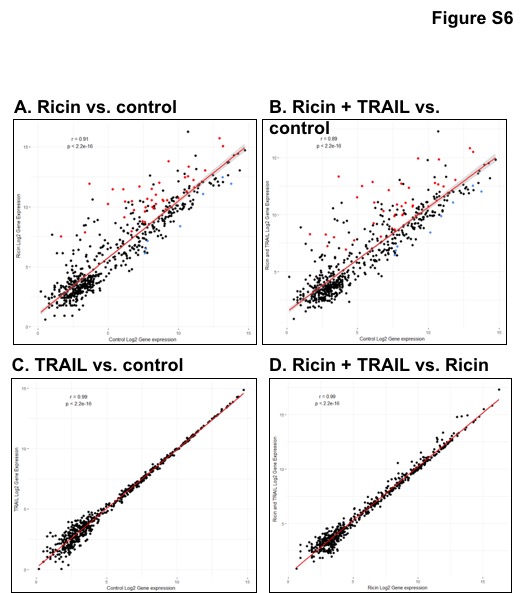

Supplement: FIG S6 [file sph005182652sf6.jpg]
