# Supplementary material for: TRAIL (CD253) Sensitizes Human Airway Epithelial Cells to Toxin-Induced Cell Death
Source: mSphere. 2018 Sep 26;3(5):e00399-18. doi: 10.1128/mSphere.00399-18 (PMC6158510; doi:10.1128/mSphere.00399-18)
Supplement: TABLE S1 [file sph005182652st1.docx]

| Supplemental Table S1 | | | | | | |
| --- | --- | --- | --- | --- | --- | --- |
|  | **Ricin + TRAIL** | | **Ricin** | | **TRAIL** | |
|  | Δ (Log 2) | *P* value | Δ (Log 2) | *P* value | Δ (Log 2) | *P* value |
| IL6 | 9.62 | 0.00966 | 8.23 | 0.0114 | -0.00165 | 1 |
| IFNB1 | 6.95 | 0.00966 | 6.11 | 0.0114 | -0.447 | 1 |
| TNF-α | 6.93 | 0.0373 | 4.99 | 0.0722 | -0.294 | 1 |
| TNFAIP3 | 6.84 | 0.00966 | 5.91 | 0.0114 | -0.102 | 1 |
| CCL20 | 6.51 | 0.00966 | 5.21 | 0.0114 | 0.438 | 1 |
| IL8 | 6.46 | 0.0302 | 5.5 | 0.0407 | 0.19 | 1 |
| ITGAM | 6.06 | 0.0324 | 5.61 | 0.0397 | -0.114 | 1 |
| CXCL11 | 5.96 | 0.0315 | 5.23 | 0.0418 | 0.0873 | 1 |
| IL1A | 5.91 | 0.00966 | 4.72 | 0.0114 | 0.139 | 1 |
| CXCL10 | 5.87 | 0.023 | 5.12 | 0.0262 | 0.24 | 1 |
| NFKBIA | 5.52 | 0.00966 | 4.44 | 0.0118 | 0.148 | 1 |
| IL29 | 5.52 | 0.0276 | 4.64 | 0.0389 | -0.858 | 1 |
| CD274 | 4.87 | 0.0439 | 4.46 | 0.0528 | 0.573 | 1 |
| NFKB2 | 4.85 | 0.00966 | 4.43 | 0.0114 | 0.0428 | 1 |
| RELB | 4.76 | 0.00966 | 4.19 | 0.0114 | 0.204 | 1 |
| CXCL2 | 4.76 | 0.0245 | 3.03 | 0.0557 | 0.0456 | 1 |
| CCL5 | 4.66 | 0.0173 | 3.8 | 0.0235 | 0.705 | 1 |
| IRAK2 | 4.5 | 0.0451 | 3.69 | 0.0687 | -0.000688 | 1 |
| KLRC3 | 4.2 | 0.0168 | 4.22 | 0.0163 | -0.266 | 1 |
| ICAM1 | 4.13 | 0.00966 | 3.02 | 0.0114 | 0.0748 | 1 |
| ICAM5 | 4.08 | 0.0451 | 2.74 | 0.0934 | 0.519 | 1 |
| BCL3 | 3.99 | 0.0248 | 3.41 | 0.0334 | -0.17 | 1 |
| CXCL1 | 3.94 | 0.00966 | 2.51 | 0.0209 | 0.0763 | 1 |
| IRF1 | 3.8 | 0.0309 | 3.17 | 0.0446 | -0.197 | 1 |
| ATG12 | 3.68 | 0.0233 | 3.74 | 0.0229 | -0.25 | 1 |
| KLRC2 | 3.58 | 0.0248 | 3.6 | 0.0235 | 0.188 | 1 |
| ETS1 | 3.57 | 0.00966 | 3.28 | 0.0114 | 0.0721 | 1 |
| IFIT2 | 3.44 | 0.0109 | 3.17 | 0.0122 | -0.0889 | 1 |
| NFKB1 | 3.4 | 0.0173 | 2.83 | 0.023 | 0.102 | 1 |
| ZEB1 | 3.03 | 0.0177 | 2.49 | 0.0235 | 0.236 | 1 |
| BCL6 | 3 | 0.0202 | 2.83 | 0.0229 | -0.244 | 1 |
| AHR | 2.95 | 0.00966 | 2.83 | 0.0114 | -0.102 | 1 |
| NFIL3 | 2.7 | 0.00966 | 2.67 | 0.0114 | 0.0501 | 1 |
| RUNX1 | 2.65 | 0.0146 | 2.48 | 0.0167 | 0.0401 | 1 |
| TICAM1 | 2.56 | 0.0355 | 1.97 | 0.0612 | 0.0505 | 1 |
| ARG2 | 2.52 | 0.00966 | 2.43 | 0.0114 | -0.0671 | 1 |
| NFATC2 | 2.41 | 0.0299 | 2.1 | 0.0391 | -0.219 | 1 |
| PLAU | 2.3 | 0.0104 | 1.89 | 0.0143 | 0.0957 | 1 |
| IL1B | 2.3 | 0.0109 | 1.36 | 0.0258 | 0.41 | 1 |
| RELA | 2.26 | 0.0126 | 2 | 0.0157 | -0.145 | 1 |
| IL6ST | 2.18 | 0.00966 | 1.91 | 0.0114 | -0.0941 | 1 |
| IL18 | 2.17 | 0.0248 | 2.13 | 0.0251 | -0.0413 | 1 |
| IFIH1 | 2.12 | 0.0307 | 1.55 | 0.0565 | -0.296 | 1 |
| TGFBR2 | 2.06 | 0.00966 | 1.92 | 0.0114 | -0.0916 | 1 |
| TRAF2 | 1.97 | 0.0345 | 1.69 | 0.0481 | 0.0253 | 1 |
| TBK1 | 1.92 | 0.00966 | 1.7 | 0.0114 | -0.0168 | 1 |
| CHUK | 1.73 | 0.0126 | 1.77 | 0.0118 | 0.0468 | 1 |
| ITGA5 | 1.73 | 0.0309 | 1.07 | 0.0779 | -0.263 | 1 |
| IL4R | 1.72 | 0.00966 | 1.47 | 0.0114 | -0.018 | 1 |
| IFNGR1 | 1.71 | 0.0177 | 1.62 | 0.0204 | -0.104 | 1 |
| ATG16L1 | 1.54 | 0.0242 | 1.78 | 0.0202 | 0.000943 | 1 |
| ATG7 | 1.37 | 0.0201 | 1.37 | 0.0204 | 0.00532 | 1 |
| RAF1 | 1.31 | 0.0242 | 1.34 | 0.023 | 0.0212 | 1 |
| CRADD | 1.24 | 0.00577 | 1.17 | 0.00613 | -0.265 | 0.942 |
| IFI16 | 1.23 | 0.0284 | 1.15 | 0.0314 | 0.0983 | 1 |
| CEBPB | 1.2 | 0.0177 | 1.03 | 0.023 | -0.0225 | 1 |
| PSMD7 | 1.14 | 0.00567 | 1.15 | 0.00508 | -0.0129 | 1 |
| HLA-B | 1.09 | 0.0355 | 0.734 | 0.0777 | -0.0724 | 1 |
| DUSP4 | 1.05 | 0.0315 | 0.69 | 0.0727 | -0.0337 | 1 |
| CD3EAP | 1 | 0.0276 | 0.811 | 0.0397 | -0.0542 | 1 |
| HLA-A | 1 | 0.0285 | 0.8 | 0.0426 | -0.0647 | 1 |
| IKBKG | 0.955 | 0.00966 | 1.04 | 0.0114 | -0.0798 | 1 |
| MAPKAPK2 | 0.892 | 0.0138 | 0.744 | 0.0203 | -0.0575 | 1 |
| ATG10 | 0.884 | 0.0304 | 0.786 | 0.0391 | -0.0256 | 1 |
| SMAD5 | 0.817 | 0.00966 | 0.786 | 0.0114 | -0.0501 | 1 |
| IRF3 | 0.731 | 0.0349 | 0.848 | 0.0262 | -0.0811 | 1 |
| C1QBP | 0.622 | 0.0238 | 0.641 | 0.0229 | 0.0507 | 1 |
| PSMC2 | 0.592 | 0.00966 | 0.673 | 0.0114 | -0.00108 | 1 |
| C14orf166 | 0.447 | 0.0373 | 0.443 | 0.0397 | -0.0431 | 1 |
| PTPN6 | -0.587 | 0.0126 | -0.627 | 0.0114 | 0.0348 | 1 |
| CD24 | -0.592 | 0.0398 | -0.771 | 0.0251 | -0.0817 | 1 |
| TAPBP | -0.648 | 0.0398 | -0.816 | 0.0262 | -0.0626 | 1 |
| TMEM173 | -0.652 | 0.0202 | -1.05 | 0.0114 | -0.08 | 1 |
| APP | -0.696 | 0.00966 | -0.743 | 0.0114 | -0.0964 | 1 |
| CD164 | -0.732 | 0.0299 | -0.748 | 0.0274 | -0.0598 | 1 |
| GPI | -0.74 | 0.00966 | -0.733 | 0.0114 | -0.0421 | 1 |
| SERPING1 | -0.769 | 0.0398 | -0.837 | 0.0367 | -0.209 | 1 |
| HLA-DMB | -0.82 | 0.0299 | -0.726 | 0.0389 | -0.145 | 1 |
| CD99 | -1.02 | 0.023 | -0.963 | 0.0235 | -0.0574 | 1 |
| BST1 | -1.23 | 0.0134 | -1.52 | 0.0114 | -0.197 | 1 |
| CD46 | -1.75 | 0.00966 | -1.84 | 0.0114 | -0.0342 | 1 |
| CD276 | -1.84 | 0.0109 | -1.85 | 0.0114 | -0.142 | 1 |
| All genes with significant changes in the mixture of ricin and TRAIL group are included, as well as the corresponding values for both the ricin and TRAIL groups. Genes are ordered from the highest log2 fold change to the lowest for the mixture of ricin and TRAIL group. | | | | | | |
